# Ultrasound Image under Artificial Intelligence Algorithm to Evaluate the Intervention Effect of Accelerated Rehabilitation Surgery Nursing on Laparoscopic Hysterectomy

**DOI:** 10.1155/2022/9042954

**Published:** 2022-03-08

**Authors:** Haiwei Yu, Ziming Zhao, Xiuping Duan, Jian Zhou, Dechun Su

**Affiliations:** ^1^Department of Obstetrics, Affiliated Hongqi Hospital of Mudanjiang Medical University, Mudanjiang 157011, Heilongjian, China; ^2^Operating Room, Affiliated Hongqi Hospital of Mudanjiang Medical University, Mudanjiang 157011, Heilongjiang, China; ^3^Department of General Surgery, Affiliated Hongqi Hospital of Mudanjiang Medical University, Mudanjiang 157011, Heilongjiang, China

## Abstract

To explore the application value of accelerated rehabilitation surgery (ERAS) nursing in laparoscopic total hysterectomy, 120 patients who underwent laparoscopic total hysterectomy for benign uterine diseases in the hospital were selected as the research object. According to different nursing schemes, they were divided into 60 cases in the experimental group (ERAS nursing program) and 60 cases in the control group (traditional perioperative nursing). All patients underwent postoperative ultrasonography, and the intraoperative and postoperative rehabilitation indexes of the two groups were analyzed. Moreover, an improved standard Capon beamforming (ISCB) algorithm is proposed, which is compared with SCB algorithm, sequential regression algorithm (SER), and recursive least square (RLS) algorithm. The results showed that the center average power and background average power (−46.92, −33.85) of the ISCB algorithm were significantly lower than those of SCB algorithm (−36.18, −23.64), SER algorithm (−39.02, −27.31), and RLS algorithm (−34.88, −24.66), while the contrast and resolution (19.11, 15.57) were significantly higher than those of SCB algorithm (12.74, 9.01), SER algorithm (13.86, 7.89), and RLS algorithm (13.26, 8.26) (*P* < 0.05). The anal exhaust time (11.84 ± 2.15 hours), analgesic effect score (3.37 ± 1.03 points), hospitalization days (3.72 ± 0.74 days), and hospitalization expenses (11859.03 ± 735.24 ¥) in the experimental group were significantly lower than those in the control group (20.95 ± 3.44 hours, 6.12 ± 1.46 points, 5.48 ± 0.91 days, 16135.22 ± 680.55 ¥) (*P* < 0.05). The score of NRS evaluation scale in the experimental group (2.28 ± 0.37) was significantly better than that in the control group (4.09 ± 0.65) (*P* < 0.05). The proportion of patients in the experimental group (very satisfied + satisfied + generally satisfied) (100%) was significantly better than that in the control group (71%), and the difference was statistically significant (*P* < 0.05). In the experimental group, there were 2 cases of postoperative fever, 1 case of nausea and vomiting, and 2 cases of lower extremity venous thrombosis. In the control group, there were 4 cases of postoperative fever, 4 cases of nausea and vomiting, and 2 cases of lower extremity venous thrombosis. In summary, ultrasound imaging based on the ISCB algorithm can display the pelvic floor structure of patients undergoing laparoscopic total hysterectomy with high quality and improve the diagnostic rate of doctors. ERAS nursing can accelerate patients' postoperative rehabilitation, reduce postoperative pain, and improve patients' satisfaction. It was worthy to be popularized and applied in the clinic.

## 1. Introduction

Pelvic floor dysfunction disease is a common health problem among women all over the world. It refers to a group of diseases caused by pelvic organ displacement and abnormal pelvic organ function caused by various factors such as weak pelvic floor support structure defect, injury, and dysfunction. Pelvic floor dysfunction disease includes pelvic organ prolapse, urinary incontinence, and sexual dysfunction [1, 2]. Total hysterectomy is one of the commonly used gynecological operations. It is often used to treat patients who have poor conservative treatment effects and no fertility requirements due to benign uterine diseases [3–5]. In addition, a total hysterectomy is also needed for the treatment of uterine malignant tumors. However, although total hysterectomy can effectively treat benign uterine diseases and some gynecological malignant diseases, it will change the anatomical position and physiological status of the whole pelvic floor tissue structure. Inevitably, it will damage the pelvic floor function [6, 7]. Accelerated rehabilitation surgery (ERAS) was systematically proposed and implemented by Danish surgeon Kehlet et al. In 2001, through the application of a series of optimization measures with evidence-based medical evidence in the perioperative period, ERAS can reduce the psychological and physiological trauma stress response of patients and promote the rapid recovery of patients [8, 9]. Currently, in the world, the ERAS nursing concept has been widely implemented as a standard scheme for perioperative treatment of colorectal diseases and gradually extended to orthopedics, gynecology, thoracic surgery, etc. [10].

Clinical imaging can be used to evaluate the recovery of patients after total hysterectomy. Magnetic resonance can reflect the pelvic floor condition of patients after treatment through multiple planes. It has the advantages of obvious soft tissue display and less interference by bone tissue, but it has high operation requirements and takes a long time [11, 12]. Radiography is simple and easy to operate, but it has poor resolution of soft tissue and ionizing radiation, so it cannot be widely used in the clinic [13]. Ultrasound can display the spatial three-dimensional relationship between tissues through static and real-time dynamic imaging of pelvic floor structure and function. It has the advantages of less time-consuming, low cost, no radiation risk, and no trauma [14, 15]. Although ultrasonic imaging has been widely used in medical clinical diagnosis, there has been no new breakthrough in the theory and method of ultrasonic imaging in recent years. The ultrasonic instruments have not been well performed in practical application in terms of imaging quality and imaging frame rate. Therefore, it is necessary to improve the imaging quality with the help of artificial intelligence technology [16]. As an ultrasonic postprocessing method, synthetic aperture focusing (SAF) ultrasonic imaging can synthesize small aperture imaging into large aperture imaging. Through point-by-point focusing, the resolution of the image does not change with position and depth, so as to obtain a higher resolution reconstructed image and provide a more reliable basis for qualitative analysis of defects [17, 18]. Therefore, this study considers to design a new ultrasonic imaging algorithm based on synthetic aperture focusing, so as to provide further help for the application of clinical ultrasonic imaging.

In summary, although there are many literature studies on the ERAS concept, its application in clinical perioperative patient care still needs to be further promoted. Therefore, 120 patients who underwent laparoscopic total hysterectomy for benign uterine diseases in the hospital were selected as the research object. They were divided into 60 cases in the experimental group (ERAS nursing scheme) and 60 cases in the control group (traditional perioperative nursing). They were all examined by ultrasound, and an improved standard Capon beamforming was proposed. The ultrasonic image is processed by the ISCB algorithm. The application value of ERAS nursing in laparoscopic hysterectomy was discussed by comparing the postoperative rehabilitation indexes between the two groups. The results showed that ERA nursing played a positive role in the prognosis of patients undergoing laparoscopic total hysterectomy. Under ERA nursing intervention, the patients recovered quickly, and the pain was relieved to the greatest extent. At the same time, under this nursing intervention, the hospitalization cost and length of stay were also better than the traditional nursing methods, and the patients' satisfaction with the whole medical process was also high. Therefore, ERA nursing was an effective nursing method, which was worthy of clinical application.

## 2. Materials and Methods

### 2.1. Research Object

In this study, 120 patients aged 30–55 years were selected as the research objects. They underwent laparoscopic total hysterectomy for benign uterine diseases in the hospital from October 2018 to May 2021. Then, according to the different nursing schemes, the patients were divided into 60 cases in the experimental group and 60 cases in the control group. The experimental group adopted the nursing scheme based on the ERAS concept, and the control group adopted the traditional perioperative nursing scheme. This study has been approved by the medical ethics committee of the hospital. The patients and their families understood the study and signed the informed consent.

Inclusion criteria were as follows: (1) the feasibility of laparoscopic hysterectomy was evaluated by doctors; (2) patients who voluntarily participated in the study and signed the informed consent; (3) no pelvic floor function recovery treatment was performed before operation.

Exclusion criteria were as follows: (1) patients with immunological diseases; (2) patients with severe cardiovascular and cerebrovascular diseases; (3) patients with liver and kidney dysfunction; (4) the uterine volume is less than 12 weeks of gestation; (5) patients with uterine malignant tumor; (6) conversion to laparotomy.

### 2.2. Nursing Methods

ERAS nursing was used in the experimental group: (1) the doctors taught the patients before operation, instructed the patients to eat normally one day before the operation, and have no water three hours before operation; (2) relevant departments conducted preoperative consultation and paid attention to preoperative visit; (3) the operating room temperature was kept at about 23°C; the infusion liquid was heated; the infusion speed was controlled; an electric blanket was laid; and the abdominal cavity was washed with 0.9% sodium chloride injection at room temperature; (4) patient-controlled intravenous analgesia pump was used for analgesia after the operation. For patients with low pain tolerance, nonsteroidal drugs were used for analgesia according to the doctor's advice; (5) normal drinking water was available two hours after the operation. The patient was given a liquid diet after waking up, changed to a semiliquid diet after ventilation, and began to eat normally one day later.

The control group used the traditional perioperative nursing methods: health education and simple visit were given to the patients before the operation. The patients were told to fast 12 hours and have no water 8 hours before the operation. The patients were given routine cleaning enema and a drainage tube placement before operation. The patient can drink water properly 6 hours after the operation and can eat liquid food after exhaust. The catheter can be removed if the drainage fluid is less than 50 mL.

### 2.3. Ultrasonic Examination Method

The ultrasonic diagnostic instrument with a probe frequency of 4-8 MHz was used. Before the examination, the patient was given a clean enema and filled the bladder, and the patient was instructed to perform standard Valsalva action and anal lifting action. During the examination, firstly, the patient took the bladder lithotomy position. Secondly, the probe was placed in the middle of the labia, gradually approached to the front wall of the vagina, and moved slowly towards the bladder neck. The probe was firmly grasped to prevent large sliding during the whole process. Thirdly, the probe was rotated and tilted to clearly show the midsagittal section of the pelvic cavity, obtain the standard sections of the anterior, middle, and posterior pelvic cavity, and measure the relevant pelvic floor ultrasound parameters. Finally, the patient was instructed to perform Valsalva action again to observe the changes of the pelvic floor structure, obtain the standard sections of anterior, middle, and posterior pelvic again, and measure the relevant ultrasonic data.

The following parameters were obtained: posterior vesicourethral angle (rRA) at rest, posterior vesicourethral angle (vRA) at maximum Valsalva, the distance between the bladder neck and posterior lower edge of the pubic symphysis (rBNSD) at rest, the distance between the bladder neck and posterior lower edge of the pubic symphysis (vBNSD) at maximum Valsalva, the distance between the posterior bladder wall and posterior lower edge of th pubic symphysis (rBLSD) at rest, the distance between the posterior wall of the bladder and the posterior lower edge of the pubic symphysis (vBLSD), the urethral inclination angle (rUTA) at rest, and the urethral inclination angle (vUTA) at maximum Valsalva.

### 2.4. Improved Standard Capon Beamforming Algorithm

The weight vector of the SCB algorithm was mainly related to interference noise covariance and guidance vector, so optimizing these two factors can improve the effect of ultrasonic imaging. In this study, a stable adaptive beamforming was proposed to optimize the interference noise covariance and steering vector to obtain the best beam output. Firstly, an ultrasonic array was set up to receive signals. The equation was as follows:

(1) $$\begin{array}{c} P\left(t\right)=H\left(\delta \right)R\left(t\right)+M\left(t\right). \end{array}$$

In (1), *P*(*t*) was the array received signal matrix, *R*(*t*) was the transmitted signal, *H*(*δ*) was the guidance matrix, *M*(*t*) was the noise signal, and *t* was the time series. After array delay reception, beamforming can finally be equations (2), (3), and (4):

(2) $$\begin{array}{c} Q\left(t\right)=Z^{E}\left(t\right)+P_{de}\left(t\right), \end{array}$$

(3) $$\begin{array}{c} P_{de}\left(t\right)=\left[p_{1}\left(t-\Psi_{1}\right),p_{2}\left(t-\Psi_{2}\right),p_{M}\left(t-\Psi_{M}\right)\right], \end{array}$$

(4) $$\begin{array}{c} Z\left(t\right)=\left[Z_{1}\left(t\right),Z_{2}\left(t\right),Z_{M}\left(t\right)\right]. \end{array}$$

In equations (2), (3), and (4), *P*_*de*_(*t*) was the signal after focusing delay, *Z*(*t*) was the weighted vector, Ψ was the array element delay, [·]^*E*^ was the conjugate device, and *M* represented the number of array elements.

In the case of a coherent source, multiple signals would cancel each other, which would affect the interference noise covariance matrix. When the matrix was decomposed, many double zero eigenvalues would appear, resulting in the loss of many useful signals. Therefore, the Toeplitz property would be used and then, correct and optimize the interference noise covariance. Then, equation (5) was obtained.

(5) $$\begin{array}{c} F_{P\left(1+m\right)}=F_{\left(1+m\right)}+K_{v}F_{\left(1+m\right)}^{\prime \prime }K_{v}. \end{array}$$

In (5), *F*_(1+*m*)_ was the interference noise covariance, [·]^″^ was the conjugate matrix, and *K*_*v*_ was the inverse identity matrix. Then, the guidance amount was optimized. In this study, a circular constraint set was introduced to constrain the projection of the guidance amount in the signal subspace, and its feature decomposition can be expressed as

(6) $$\begin{array}{c} F_{P\left(1+m\right)}=W_{s}\Pi_{s}W_{s}+W_{m}\Pi_{m}W_{m}^{E}. \end{array}$$

In (6), Π_*s*_ was the eigenvalue of the target signal and interference signal, Π_*m*_ was the eigenvalue of the noise signal, *W*_*s*_ was the feature space composed of the feature vector corresponding to the larger eigenvalue, and *W*_*m*_ was the feature space composed of the feature vector corresponding to the smaller eigenvalue. An appropriate amount of guidance can be described as a quadratic constraint problem.

(7) $$\begin{array}{c} \left\{\begin{array}{cc} \underset{\lambda }{\min} & {\left(W_{s}W_{s}^{E}\lambda \right)}^{E}F_{P\left(1+m\right)}\left(W_{s}W_{s}^{E}\lambda \right),\\ s.t. & {\left\|\lambda -\bar{\lambda }\right\|}^{2}\leq \tau . \end{array}\right) \end{array}$$

In (7), *W*_*s*_*W*_*s*_^*E*^*λ* was the projection of the guidance vector in the signal subspace, *τ* was the error constant, and $\bar{\lambda }$ was the preset guidance vector. By substituting (6) into equation (7), equation (8) was obtained.

(8) $$\begin{array}{c} \left\{\begin{array}{cc} \underset{\lambda }{\min} & \lambda^{E}W_{s}\Pi_{s}W_{s}^{E}\lambda ,\\ s.t. & {\left\|\lambda -\bar{\lambda }\right\|}^{2}\leq \tau . \end{array}\right) \end{array}$$

Then, the above equation was solved by Lagrange, and equation (9) was obtained.

(9) $$\begin{array}{c} G\left(\beta \right)=\lambda^{E}W_{s}\Pi_{s}W_{s}^{E}\lambda +\beta \left[{\left(\lambda -\bar{\lambda }\right)}^{E}\left(\lambda -\bar{\lambda }\right)-\tau \right]. \end{array}$$

By deriving *λ*, equation (10) was obtained.

(10) $$\begin{array}{c} \lambda =\bar{\lambda }\cdot {\left(K+\frac{W_{s}\Pi_{s}W_{s}^{E}}{\lambda }\right)}^{-1}. \end{array}$$

In (10), *β* was the Lagrange operator. The size of the signal subspace of the interference noise covariance also needed to be discussed. If the subspace was too small, some expected signals would be omitted, and if the subspace was too large, it would contain noise signals. Therefore, the threshold value in this study was set to 0.5 times the maximum eigenvalue, and the eigenvalue greater than the threshold value can form the signal subspace. By optimizing the interference noise covariance and guidance vector, this study constructed the final improved standard Capon beamforming (ISCB) algorithm.

### 2.5. Observation Indicators

The experimental observation indexes were as follows.

1. Operation time, intraoperative bleeding, and anal exhaust time
2. Postoperative analgesic effect, postoperative complications, average hospital stay, average hospital stay, and medical expenses
3. The postoperative analgesic effect was evaluated by numerical rating scale (NRS); Newcastle Satisfaction with Nursing Scales (NSNS) was used to evaluate patients' nursing satisfaction

NRS evaluation scale: It was composed of 11 numbers from 0 to 10. 11 numbers from 0 to 10 were used by patients to describe the intensity of pain. The larger the number was, the more severe the pain was. Evaluation criteria were as follows: 0: painless; 1–3: moderate pain (pain does not affect sleep); 4–6: moderate pain; 7–9 severe pain (unable to sleep or wake up from sleep); 10: sharp pain.

NSNS scale: It is a scale to investigate patients' satisfaction with nursing services in the hospital (ward). 1 = very dissatisfied, 2 = dissatisfied, 3 = average, 4 = satisfied, 5 = very satisfied.  Table 1 below illustrates the specific scoring entries.

### 2.6. Expression of Average Power

The expression of average power referred to the power consumed by the resistance part of the circuit, which was expressed in the letter P, in Watts. The average value of power, apparent power, and instantaneous power in a cycle was called average power. Average power was the power actually consumed, which was also called active power. The following equation (11) was used to express it.

(11) $$\begin{array}{c} P=\frac{1}{T}\int_{0}^{T}p\left(t\right)\text{d}t. \end{array}$$

### 2.7. Statistical Methods

The data of this study were analyzed by SPSS19.0 statistical software. The measurement data were expressed by mean ± standard deviation (‾*x* ± *s*), and the counting data were expressed by percentage (%). One-way analysis of variance was used for pairwise comparison. The difference was statistically significant (*P* < 0.05).

## 3. Results

### 3.1. Imaging Performance of ISCB Algorithm

In this study, the SCB algorithm, SER algorithm, RLS algorithm, and ISCB algorithm were introduced to compare the imaging performance (center average power, background average power, contrast, and resolution).

**Figure 1:**
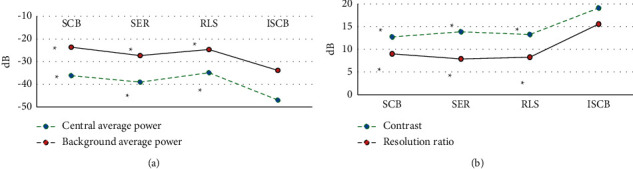
Imaging performance analysis of the ISCB algorithm. (a) Center average power and background average power. (b) Contrast and resolution. *∗* indicates that the difference was statistically significant compared with the ISCB algorithm (*P* < 0.05).

Figure 1 suggests that the center average power and background average power of the ISCB algorithm were remarkably lower than those of the SCB algorithm, SER algorithm, and RLS algorithm (*P* < 0.05). The contrast and resolution of the ISCB algorithm were greatly higher than the SCB algorithm, SER algorithm, and RLS algorithm (*P* < 0.05).

**Figure 2:**
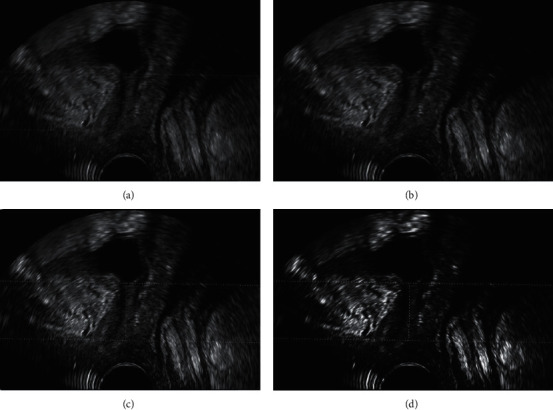
Ultrasound imaging of pelvic floor under various algorithms. (a) SCB algorithm. (b) SER algorithm. (c) RLS algorithm. (d) ISCB algorithm.

Figure 2 shows the ultrasonic imaging images under each algorithm. It indicated that the pelvic floor ultrasonic imaging images under the four algorithms can clearly display the detrusor thickness. Among them, the ISCB algorithm has few ultrasonic image artifacts and low side lobes, and the overall quality is better than the SCB algorithm, SER algorithm, and RLS algorithm.

### 3.2. Baseline Data Comparison

**Figure 3:**
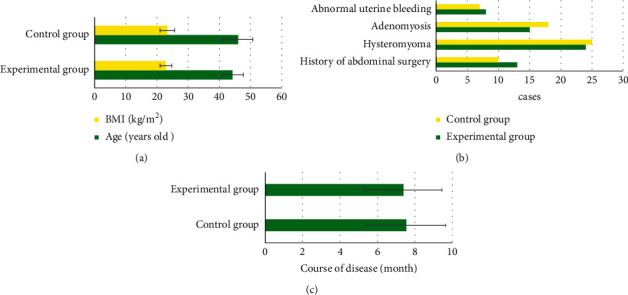
Comparison of baseline data between the two groups. (a) Age and BMI; (b) history of abdominal surgery, hysteromyoma, adenomyosis, and abnormal uterine bleeding; (c) duration of disease.

According to the comparison of baseline data between the two groups, there was no significant difference in age, BMI, history of abdominal surgery, hysteromyoma, adenomyosis, abnormal uterine bleeding, and course of disease between the two groups (*P* > 0.05).  Figure 3 illustrates the details.

### 3.3. Image Data of Some Cases

**Figure 4:**
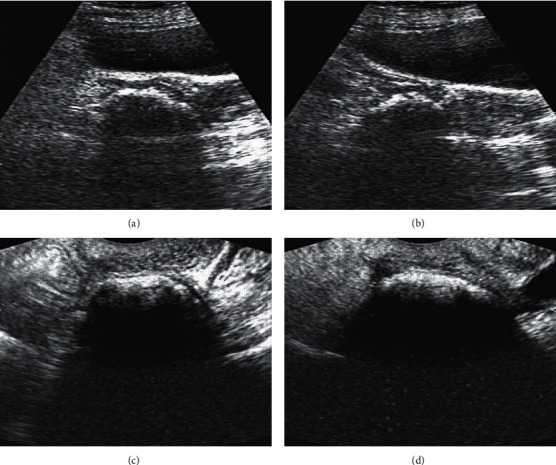
Postoperative ultrasound images of a 42-year-old patient undergoing total hysterectomy. ((a), (b)) Pelvic cavities; ((c), (d)) transvaginal uterus.

Figure 4 shows the postoperative ultrasonic image data of a 42-year-old patient. The patient underwent a subtotal hysterectomy for multiple uterine fibroids. A small amount of vaginal fluid (light yellow and light red) was found after the operation. Ultrasonography showed a strong echo mass at the upper right of the cervical internal opening, followed by wide and thick sound attenuation.

**Figure 5:**
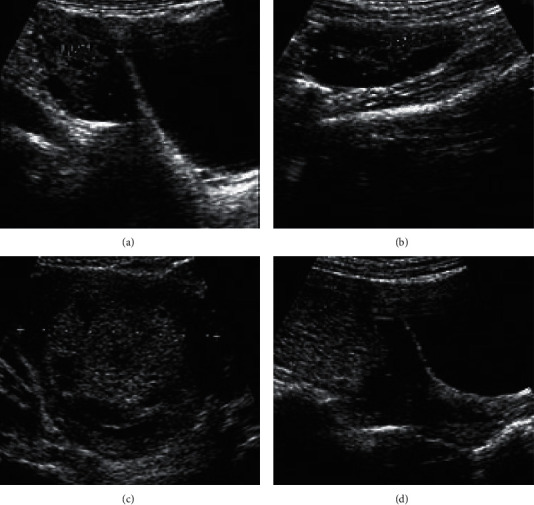
Postoperative ultrasound images of a 37-year-old patient undergoing total hysterectomy. ((a), (b)) Transabdominal; ((c), (d)) transvaginal uterus.

Figure 5 reveals the postoperative ultrasonic image data of a 37-year-old patient. Due to subtotal resection of hysteromyoma, a heterogeneous low echo mass can be seen in front of the cervix, with clear boundary, regular shape, complete capsule, uneven internal echo, and no abnormality of the cervix.

### 3.4. Comparison of Intraoperative Indexes between the Two Groups

**Figure 6:**
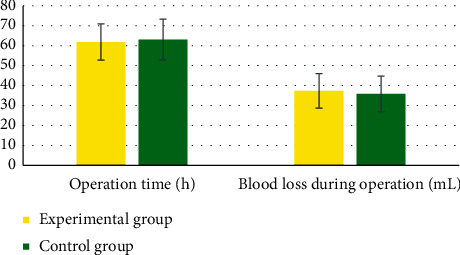
Comparison of intraoperative indexes (operation time and intraoperative bleeding) between the two groups.

Figure 6 gives the comparison of intraoperative indexes between the two groups. There was no significant difference in operation time and intraoperative bleeding between the experimental group and the control group (*P* > 0.05).

### 3.5. Comparison of Postoperative Rehabilitation Indexes between the Two Groups

**Figure 7:**
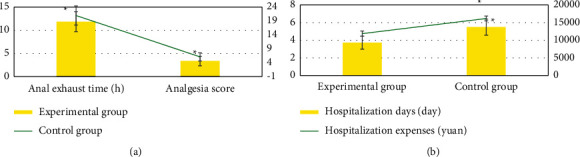
Comparison of postoperative rehabilitation indexes between the two groups. (a) Score of anal exhaust time and analgesic effect. (b) Length of stay and hospitalization expenses. *∗* indicates that there was an observable difference compared with the experimental group (*P* < 0.05).

Figure 7 indicates the comparison of postoperative rehabilitation indexes between the two groups. It can be seen that the anal exhaust time, analgesic effect score, hospitalization days, and hospitalization expenses of the experimental group were significantly lower than those of the control group (*P* < 0.05).

### 3.6. Comparison of Postoperative Complications, Nursing Satisfaction, and NRS Pain Score between the Two Groups

**Figure 8:**
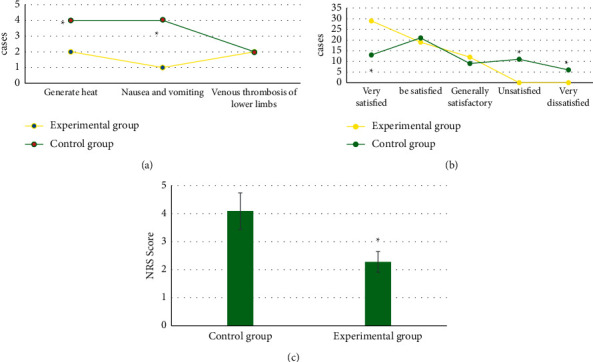
Comparison of postoperative complications, nursing satisfaction, and NRS pain score between the two groups. (a) Postoperative complication. (b) Postoperative nursing satisfaction. (c) NRS pain score. *∗* indicates that there was a significant difference compared with the experimental group (*P* < 0.05).

Figure 8(a) suggests that in the experimental group, there were 2 cases of postoperative fever, 1 case of nausea and vomiting, and 2 cases of lower extremity venous thrombosis. In the control group, there were 4 cases of postoperative fever, 4 cases of nausea and vomiting, and 2 cases of lower extremity venous thrombosis. The number of postoperative fever, nausea, and vomiting in the experimental group was significantly lower than that in the control group (*P* < 0.05).

Figure 8(b) indicates that in the experimental group, 29 patients were very satisfied, 19 were satisfied, 12 were generally satisfied, 0 were dissatisfied, and 0 were very dissatisfied. In the control group, 13 cases were very satisfied, 21 cases were satisfied, 9 cases were generally satisfied, 11 cases were dissatisfied, and 6 cases were very dissatisfied. The number of very satisfactory cases in the experimental group was remarkably higher than that in the control group (*P* < 0.05). The number of dissatisfied and very dissatisfied patients in the experimental group was obviously less than that in the control group (*P* < 0.05).

Figure 8(c) indicates that the NRS evaluation scale score of the experimental group (2.28 ± 0.37 points) was significantly better than that of the control group (4.09 ± 0.65 points), and the difference was statistically significant (*P* < 0.05).

### 3.7. Comparison of Pelvic Floor Ultrasonic Parameters between the Two Groups

**Figure 9:**
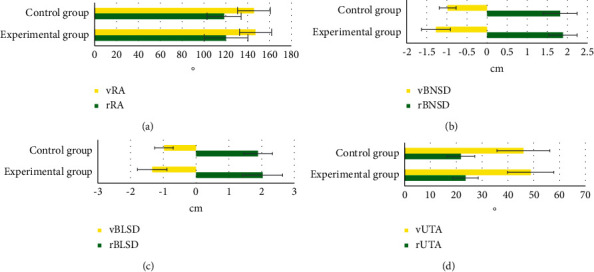
Comparison of pelvic floor ultrasound parameters between the two groups. (a) rRA and vRA. (b) rBNSD and vBNSD. (c) rBLSD and Vblsd. (d) rUTA and vUTA.

Figure 9 shows the comparison of pelvic floor ultrasound parameters between the two groups. There was no significant difference in ultrasound parameters rRA, vRA, rBNSD, vBNSD, rBLSD, vBLSD, rUTA, and vUTA between the experimental group and the control group (*P* > 0.05).

## 4. Discussions

Pelvic floor diseases usually do not show a single set of symptoms. They are usually complicated symptoms. Sometimes, they only show vague backache or lower abdominal discomfort. They also show obvious symptoms, such as urinary incontinence, pelvic floor prolapses, bladder retention symptoms, and sensory impairment (sensory symptoms). From the perspective of evidence-based medicine, the core of accelerated rehabilitation surgery is to emphasize the diagnosis and treatment concept of serving patients, optimize clinical paths, promote patients' rehabilitation, and improve patients' satisfaction with medical treatment, so as to be widely used in perioperative patient care [19, 20]. Ultrasound is a commonly used imaging method in the clinic in recent years. It can evaluate pelvic floor diseases and postoperative rehabilitation in multiple aspects. To further improve the effect of ultrasonic diagnosis, an improved ISCB algorithm is proposed and compared with the traditional algorithm. The results show that the center average power and background average power of the ISCB algorithm are significantly lower than those of the SCB algorithm, SER algorithm, and RLS algorithm. However, the contrast and resolution of the ISCB algorithm are just the opposite, and the difference is significant (*P* < 0.05). It indicates that the ISCB algorithm successfully corrects the error of guidance vector and performs well in anti-interference, imaging contrast, and resolution [21]. Compared with the pelvic floor ultrasound imaging effect, the ISCB algorithm has few ultrasound image artifacts and low side lobes, and the overall quality is better than the SCB algorithm, SER algorithm, and RLS algorithm, which corresponds to the above quantitative data results. Through experimental verification, compared with other algorithms, the improved standard Capon beamforming (ISCB) algorithm had significantly improved the spatial resolution of imaging, improved the contrast of imaging, and simplified the complexity of the algorithm to a certain extent. Then, 120 patients who underwent laparoscopic total hysterectomy for benign uterine diseases in our hospital were selected as the research object. According to different nursing schemes, they were divided into 60 cases in the experimental group (ERAS concept nursing scheme) and 60 cases in the control group (traditional perioperative nursing scheme). The intraoperative and postoperative rehabilitation indexes of the two groups were analyzed. Firstly, it was revealed that the anal exhaust time of patients in the experimental group was significantly lower than that in the control group, and the difference was statistically significant (*P* < 0.05). This suggests that unconventional preoperative mechanical intestinal preparation in ERAS nursing can reduce the stress stimulation response of perioperative patients, especially elderly patients, and avoid discomfort such as dehydration and electrolyte imbalance [22]. The hospitalization days and hospitalization expenses of the experimental group were greatly lower than those of the control group (*P* < 0.05), and the difference was statistically significant (*P* < 0.05). This is because the way of shortening preoperative fasting time in ERAS nursing can help patients reduce postoperative insulin resistance, shorten postoperative hospital stay, and reduce hospital cost. The postoperative analgesic effect score of the patients in the experimental group was significantly lower than that in the control group. However, the satisfaction in the experimental group was higher than that in the control group, and the difference was statistically significant (*P* < 0.05), which was similar to the research results of Athanasiou et al. (2020) [23]. In ERAS nursing, the postoperative analgesia pump can effectively alleviate the pain feeling of the patients, reduce the unpleasant experience of the patients, accelerate the recovery of the patients, and improve patients' satisfaction with the medical process. Postoperative follow-up showed that in the experimental group, there were 2 cases of postoperative fever, 1 case of nausea and vomiting, and 2 cases of lower extremity venous thrombosis. In the control group, there were 4 cases of postoperative fever, 4 cases of nausea and vomiting, and 2 cases of lower extremity venous thrombosis. It can be clearly seen that the number of postoperative complications in the experimental group is less than that in the control group. This shows that ERAS nursing can have a positive impact on the prognosis of patients undergoing laparoscopic total hysterectomy and reduce the occurrence of concurrent symptoms [24, 25].

## 5. Conclusion

In this study, two groups of patients undergoing laparoscopic total hysterectomy were tested with different nursing methods, and the intraoperative and postoperative rehabilitation indexes of the two groups were analyzed. The results showed that ultrasound imaging based on the artificial intelligence algorithm can display the pelvic floor structure of patients undergoing laparoscopic total hysterectomy with high quality and help doctors to make an accurate evaluation. ERAS nursing can have a positive impact on the prognosis of patients undergoing laparoscopic total hysterectomy. It can remarkably accelerate their postoperative rehabilitation, relieve their pain, reduce their hospitalization expenses, and improve their satisfaction with the whole medical process. Thus, it is worthy of clinical promotion. The results provided a data reference for the popularization and application of ERAS in perioperative patient care. However, the number of patients with laparoscopic total hysterectomy in this study is small, and the prognosis of patients with different uterine diseases is not compared. In the follow-up, more patient samples will be considered to further explore whether ERAS nursing will have a different impact on patients with different uterine diseases.

## Figures and Tables

**Table 1:** Newcastle nursing service satisfaction scale (NSNS).

Order number	TEST item	Very dissatisfied	Dissatisfied	Commonly	Satisfied	Very satisfied
1	The time spent by the nurse for you	1	2	3	4	5
2	Working competence of nurses	1	2	3	4	5
3	There will always be nurses around you when you need them	1	2	3	4	5
4	How well the nurses know about your care	1	2	3	4	5
5	How fast the nurses handle your call	1	2	3	4	5
6	The way the nurse treats you makes you feel at home	1	2	3	4	5
7	The amount of disease and treatment information provided by the nurse	1	2	3	4	5
8	Number of times nurses visited the ward	1	2	3	4	5
9	Help provided by nurses	1	2	3	4	5
10	The way the nurse explains the problem for you	1	2	3	4	5
11	How much the nurse reassures your relatives or friends	1	2	3	4	5
12	Nurses' attitude towards their own work	1	2	3	4	5
13	The type of disease and treatment information the nurse provides you	1	2	3	4	5
14	How much respect the nurses have for you in the nursing process	1	2	3	4	5
15	The way the nurse listens to your troubles and concerns	1	2	3	4	5
16	On the premise of abiding by the rules and regulations, the nurse gives you the degree of freedom during hospitalization	1	2	3	4	5
17	Willingness of the nurse to respond to your request	1	2	3	4	5
18	How much privacy the nurses protect you	1	2	3	4	5
19	The nurse can understand your needs	1	2	3	4	5

## Data Availability

The data used to support the findings of this study are available from the corresponding author upon request.

## References

[1] Yilmaz G., Akça A., Aydin N. (2018). Enhanced recovery after surgery (ERAS) versus conventional postoperative care in patients undergoing abdominal hysterectomies. *Ginekologia Polska*.

[2] Lv Z., Xiu W. (2020). Interaction of edge-cloud computing based on SDN and NFV for next generation IoT. *IEEE Internet of Things Journal*.

[3] Marchand G. J., Azadi A., Sainz K. (2021). The efficacy of acetaminophen in ERAS protocols for total laparoscopic hysterectomy. *Journal of the Society of Laparoendoscopic Surgeons*.

[4] Chen Y., Hu S., Mao H., Deng W., Gao X. (2020). Application of the best evacuation model of deep learning in the design of public structures. *Image and Vision Computing*.

[5] Street A. D., Elia J. M., McBroom M. M. (2020). The impact of implementation of a hysterectomy enhanced recovery pathway on anesthetic medication costs. *Journal of Comparative Effectiveness Research*.

[6] Nazzani S., Preisser F., Mazzone E. (2018). In-hospital length of stay after major surgical oncological procedures. *European Journal of Surgical Oncology*.

[7] Renaud M.-C., Bélanger L., Lachapelle P., Grégoire J., Sebastianelli A., Plante M. (2019). Effectiveness of an enhanced recovery after surgery program in gynaecology oncologic surgery: a single-centre prospective cohort study. *Journal of Obstetrics and Gynaecology Canada*.

[8] Wu Y. J., Liu W.-J., Yuan C.-H. (2020). A mobile-based barrier-free service transportation platform for people with disabilities. *Computers in Human Behavior*.

[9] Ebner F., Schulz S. V. W., de Gregorio A. (2018). Prehabilitation in gynecological surgery? What do gynecologists know and need to know. *Archives of Gynecology and Obstetrics*.

[10] Ying X., Zhang Y., Yu M. (2020). Cascade marker removal algorithm for thyroid ultrasound images. *Medical, & Biological Engineering & Computing*.

[11] Wijntjes J., Alfen N. (2021). Muscle ultrasound: present state and future opportunities. *Muscle & Nerve*.

[12] Baloescu C., Toporek G., Kim S. (2020). Automated lung ultrasound B-line assessment using a deep learning algorithm. *IEEE Transactions on Ultrasonics, Ferroelectrics, and Frequency Control*.

[13] Brattain L. J., Telfer B. A., Dhyani M., Grajo J. R., Samir A. E. (2018). Machine learning for medical ultrasound: status, methods, and future opportunities. *Abdominal Radiology*.

[14] Bharadwaj S., Almekkawy M. Faster search algorithm for speckle tracking in ultrasound images.

[15] Lyu J., Ling S. H., Banerjee S. 3D ultrasound spine image selection using convolution learning-to-rank algorithm.

[16] Rinaldi L., Milione S., Fascione M. C. (2020). Relevance of lung ultrasound in the diagnostic algorithm of respiratory diseases in a real-life setting: a multicentre prospective study. *Respirology*.

[17] Xie S., Yu Z., Lv Z. (2021). Multi-disease prediction based on deep learning: a survey. *Computer Modeling in Engineering and Sciences*.

[18] Nazzani S., Bandini M., Preisser F. (2019). Postoperative paralytic ileus after major oncological procedures in the enhanced recovery after surgery era: a population based analysis. *Surgical Oncology*.

[19] Chung G., Hinoul P., Coplan P., Yoo A. (2021). Trends in the diffusion of robotic surgery in prostate, uterus, and colorectal procedures: a retrospective population-based study. *Journal of Robotic Surgery*.

[20] Scheib S. A., Thomassee M., Kenner J. L. (2019). Enhanced recovery after surgery in gynecology: a review of the literature. *Journal of Minimally Invasive Gynecology*.

[21] Ellis D. B., Agarwala A., Cavallo E. (2021). Implementing ERAS: how we achieved success within an anesthesia department. *BMC Anesthesiology*.

[22] Weston E., Noel M., Douglas K. (2020). The impact of an enhanced recovery after minimally invasive surgery program on opioid use in gynecologic oncology patients undergoing hysterectomy. *Gynecologic Oncology*.

[23] Athanasiou S., Zacharakis D., Grigoriadis T. (2020). Vaginal hysterectomy with anterior and posterior repair for pelvic organ prolapse under local anesthesia: results of a pilot study. *International Urogynecology Journal*.

[24] Cohen R., Gooberman-Hill R. (2019). Staff experiences of enhanced recovery after surgery: systematic review of qualitative studies. *BMJ Open*.

[25] Keil D. S., Schiff L. D., Carey E. T. (2019). Predictors of admission after the implementation of an enhanced recovery after surgery pathway for minimally invasive gynecologic surgery. *Anesthesia & Analgesia*.

